# An efficient system to generate monoclonal antibodies against membrane-associated proteins by immunisation with antigen-expressing mammalian cells

**DOI:** 10.1186/1472-6750-10-87

**Published:** 2010-12-15

**Authors:** Anita M Dreyer, Jeremy Beauchamp, Hugues Matile, Gerd Pluschke

**Affiliations:** 1 Molecular Immunology Unit, Swiss Tropical and Public Health Institute, Socinstrasse 57, CH-4002 Basel, Switzerland; 2 University of Basel, Petersplatz 1, CH-4003 Basel, Switzerland; 3 Pharma Research Basel, F. Hoffmann-La Roche Ltd., Grenzacherstrasse 124, CH-4070 Basel, Switzerland

## Abstract

**Background:**

The generation of monoclonal antibodies specific for protein antigens usually depends on purified recombinant protein for both immunisation and hybridoma screening. Purification of recombinant protein in sufficient yield and purity is a tedious undertaking and can be demanding especially in the case of membrane proteins. Furthermore, antibodies generated against a purified recombinant protein are frequently incapable of binding to the endogenous protein in its native context.

**Results:**

We describe a strategy to generate monoclonal antibodies against membrane or membrane-associated proteins that completely bypasses any need for purified recombinant antigen. This approach utilises stably transfected mammalian cells expressing recombinant antigens on their cell surface for immunisation of mice. The transfected cells are also used for measuring seroconversion, hybridoma selection and antibody characterisation. By presenting the antigen in its native conformation for immunisation and hybridoma selection, this procedure promotes the generation of antibodies capable of binding to the endogenous protein. In the present study, we applied this approach successfully for three predicted GPI-anchored proteins of the malaria parasite *Plasmodium falciparum*.

**Conclusions:**

The described entirely cell-based technology is a fast and efficient approach for obtaining antibodies reactive with endogenous cell-surface proteins in their native conformation.

## Background

Since the development of the B-cell hybridoma technology for the generation of monoclonal antibodies (mAbs) in 1975 by Kohler and Milstein [1], mAbs have become molecular tools of great value. Due to their high specificity, mAbs are used throughout biology for the characterisation of protein function and distribution. Besides their usage in research, mAbs are also widely utilised as diagnostic and therapeutic agents [2,3]. Due to this wide range of applications the generation of mAbs became a standard procedure. However the generation of mAbs against protein antigens can still be problematic, since for studies in physiological settings, it is important that the mAbs recognise the target protein in its native conformation.

Frequently, mAbs are raised against synthetic peptides derived from the predicted sequence of the target protein. Unfortunately, these Abs, though strongly reactive with peptide, frequently fail to recognise the native protein [4]. Another standard procedure to generate mAbs uses purified recombinantly expressed proteins. Prokaryotic expression systems are the most widely used expression hosts. But when studying mammalian surface proteins it is often necessary to use mammalian expression systems, as they are more likely to produce functional proteins with the appropriate disulfide-bonds and posttranslational modifications [5,6]. Although introduction of affinity tags simplifies purification, it often remains difficult to obtain recombinant protein in native conformation and in sufficient yield and purity. This applies most notably to membrane and membrane-associated proteins, as they are likely to lose their native structure during the purification processes [7].

When attempting to generate mAbs capable of recognising the native protein, it is also critical to use the target protein in its native conformation not only in the immunisation step but also for the screening procedure. Many standard hybridoma-screening protocols make use of recombinant proteins immobilized on solid supports, which may significantly alter protein conformation [8].

With the objective of generating mAbs specifically recognising membrane-associated proteins in their native conformation, we applied a methodology that bypasses any need for purified recombinant protein. This strategy utilises antigens expressed on the surface of stably transfected mammalian cells both for immunisation of mice and for immunoassays, such as testing seroconversion, hybridoma selection and mAb characterisation.

In the present study, we applied this approach for three predicted GPI-anchored proteins of *Plasmodium falciparum*. *P. falciparum *is the causative agent of malaria tropica, which claims 300-600 million clinical cases and more than 2 million deaths each year [9]. Malaria is transmitted to humans by the bite of an infected female *Anopheles *mosquito. The inoculated sporozoites infect hepatocytes where the parasites undergo schizogony resulting in the rupture of the infected liver cell and release of free merozoites, which infect erythrocytes. Upon intra-erythrocytic schizogony red blood cells rupture and release more merozoites. Some of these differentiate into gametocytes, which, when taken up by a feeding mosquito bring about the sexual cycle, resulting in the development of sporozoites located in the salivary gland of the mosquito. Highly specific cell-cell interactions between the invasive forms of the parasite and the corresponding host cells are pivotal steps in the complex life cycle of *P. falciparum*, which depend on specific molecular interactions of cell surface molecules. Being exposed to potentially parasite inhibitory antibodies makes parasite proteins involved in cell-cell interactions of particular interest with respect to vaccine development. Most proteins that coat the surface of the extracellular forms of *P. falciparum *are known or presumed to be GPI anchored [10]. We anticipated the generation of mAbs against hypothetical proteins of *P. falciparum *predicted to be a GPI-anchored membrane protein in order to get a tool to characterize their properties and potential as vaccine candidate antigens. Based on being predicted to be expressed in invasive stages and to contain a GPI-anchor motif, three hypothetical proteins were selected: *PFF0620c, PFD1130w *and *PF14_0325*. For *PFD1130w *and *PF14_0325 *published transcriptional analysis data showed elevated expression levels in late stages of the asexual blood stage cycle [11,12]. For both predicted proteins no function can currently be assigned as no significant homology to functional domains of other characterized gene products could be identified. *PFF0620c *contains two S48-45 sexual stage antigen-like domains, which are characterized by the presence of 6 cysteines. Other members of this protein family have been shown to be located on the surface of the parasite and some of them are known to play a role in cell-cell interactions [13]. Mass spectrometry data indicate expression of *PFF0620c *at the sporozoite stage [14].

To achieve surface localisation of the recombinant *P. falciparum *proteins on mammalian cells despite potential differences of the secretory machinery between *Plasmodium *and host cells, the *P. falciparum *coding sequences were altered in several ways. The endogenous secretion signal sequences and GPI-attachment sites were replaced with the signal peptide of bee venom melittin and the transmembrane domain of mouse glycophorine A, respectively. To allow assessment of surface localization, antibody-tags were introduced on both sides of the transmembrane domain.

We expect that this entirely cell-based system, capable of generating mAbs against membrane-associated antigens, is applicable for a wide range of cell-surface proteins.

## Results

### Expression of predicted GPI-anchored *P. falciparum *proteins on the cell surface of mammalian cells

**Figure 1:**
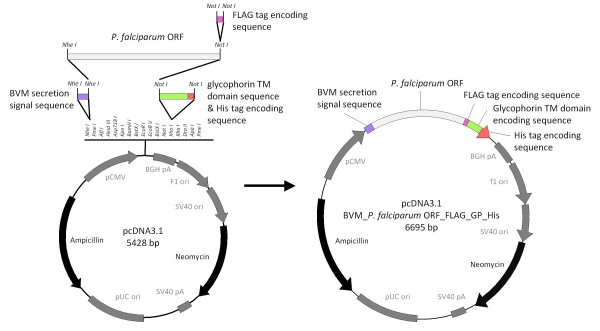
**Construction of expression plasmids**. Schematic diagram of pcDNA3.1 BVM_PFF0620C_FLAG_GP_His, pcDNA3.1 BVM_PFD1130W_FLAG_GP_His and pcDNA3.1 BVM_PF14_0325_FLAG_GP_His (pcDNA3.1 BVM_*P. falciparum *ORF_FLAG_GP_His). The commercially available pcDNA3.1 expression plasmid (Invitrogen) was modified to contain chimeric genes consisting of the secretion signal sequence of bee venom melittin, the codon-optimised sequence encoding the *P. falciparum *proteins PFF0620C, PFD1130W or PF14_0325 without secretion signal sequence and GPI-attachment signal sequence, a FLAG tag encoding sequence, a sequence encoding the transmembrane domain of mouse glycophorin-A and a His tag encoding sequence.

**Figure 2:**
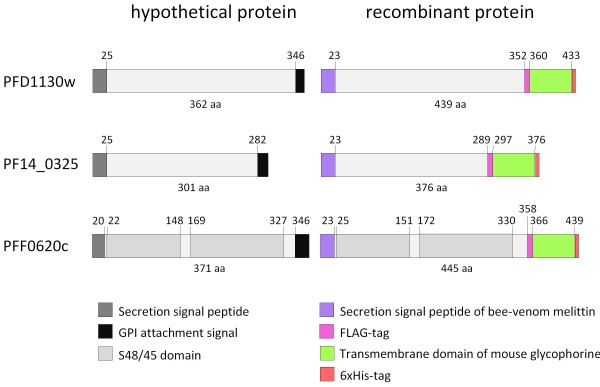
**Schematic representation of the modified hypothetical *P. falciparum *proteins PFF0620C, PFD1130W or PF14_0325**. The hypothetical *P. falciparum *proteins PFF0620C, PFD1130W or PF14_0325 (left), and their modification for ectopic expression (right) are schematically depicted. The diagram shows the location of the secretion signal peptide, the GPI-attachment signal sequence, PFAM predicted domains, FLAG tag, hexa-His tag and transmembrane domains. Respective amino acid positions of domains are indicated.

The three predicted open reading frames (ORFs) PFF0620c, PFD1130w and PF14_0325 of *P. falciparum *were expressed on the cell surface of HEK cells using the expression plasmids pcDNA3.1_BVM_PFF0620c_FLAG_GLP_His, pcDNA3.1_BVM_PFD1130W_FLAG_GLP_His and pcDNA3.1_BVM_PF14_0325_FLAG_GLP_His, respectively (Figure 1). To ensure expression on the cell surface, the predicted ORFs were modified in several ways: i. the endogenous *P. falciparum *sequences were codon-optimised for expression in mammalian cells; ii. the endogenous secretion signal sequences were replaced by the secretion signal sequence of bee-venom melittin; iii. for membrane anchoring the transmembrane domain encoding sequence of mouse glycophorin was used instead of the predicted GPI-attachment signal sequences; iv. to allow expression analysis, a FLAG tag was inserted N-terminally of the transmembrane domain and a hexa-His tag was placed at the C-terminus. The two tags were positioned just before and after the transmembrane domain to facilitate verification of the extracellular localisation of the recombinantly expressed malaria antigens (Figure 2 and additional files 1 and 2).

HEK-derived cell lines expressing *P. falciparum *PFF0620c, PFD1130W and PF14_0325 were established by stable transfection. Expression of recombinant proteins was confirmed by Western blot analysis using anti-His tag and anti-FLAG tag antibodies. While protein bands appeared when lysates of the three stably transfected cell lines were analysed, no staining was obtained with non-transfected HEK cells (data not shown). Also in immune fluorescence analysis (IFA) transfectants showed strongly increased staining both with anti-His tag and with anti-FLAG tag antibodies (data not shown).

**Figure 3:**
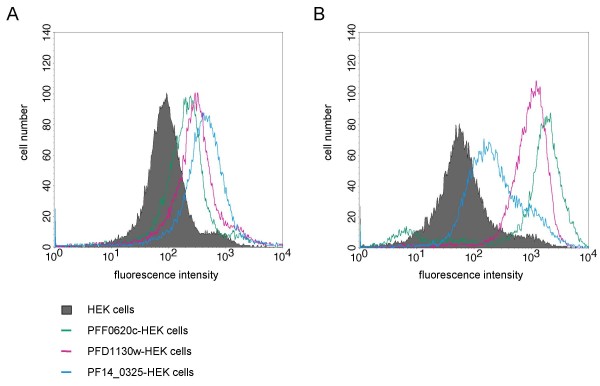
**Detection of the *P. falciparum *proteins PFF0620C, PFD1130W or PF14_0325 displayed on stably transfected HEK cells**. Histogram plots show flow-cytometric analysis of non-transfected and high-expressing cell pools of transfected HEK cells (PFF0620C-HEK, PFD1130W-HEK, PF14_0325-HEK) stained with anti-His tag antibody (A) or anti-FLAG tag antibody (B).

To obtain highly expressing cell lines, transfectants were separated into high-expressing cell-pools by fluorescent-activated-cell-sorting after surface staining with anti-FLAG tag antibodies. After several days in culture the mean fluorescence intensities of the high-expressing cell pools of the transfectants PFF0620c-HEK, PFD1130W-HEK and PF14_0325-HEK, were ten, seven and three times higher compared to non-transfected HEK cells (Figure 3B).

**Figure 4:**
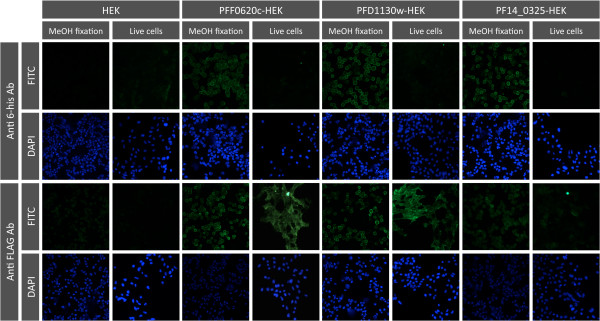
**Cell-surface expression of PFF0620C, PFD1130W or PF14_0325 on stably transfected HEK cells**. Fluorescence staining of living or methanol fixed untransfected HEK cells, PFF0620C-HEK cells, PFD1130w-HEK cells and PF14_0325 HEK cells after staining with anti-His tag or anti-FLAG tag antibodies and FITC-labelled anti-mouse IgG antibodies. Nuclei were stained with DAPI. With the anti-FLAG tag antibody both living and methanol-fixed transfectants were stained, whereas the anti-His tag antibody only stained methanol-fixed transfectants, indicating intracellular localisation of the His tag and extracellular localisation of the FLAG tag together with the *P. falciparum *derived protein domain.

Live-cell staining of PFF0620c-HEK, PFD1130W-HEK or PF14_0325-HEK cells with anti-FLAG tag antibody yielded strong signals. In contrast, staining with anti-His tag antibody gave strong signals on methanol fixed cells but not on living cells (Figure 4). From these results we deduced that PFF0620c, PFD1130W and PF14_0325 were expressed and anchored in the cell membrane with the FLAG tag located extracellularly and the His tag intracellularly.

### Development of malaria antigen specific antibodies in mice immunised with transfected HEK cells

**Figure 5:**
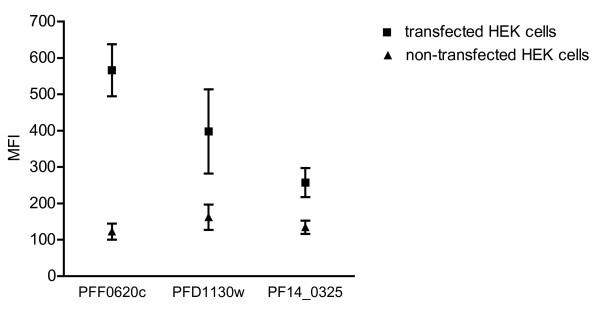
**Staining of transfected HEK cells by serum antibodies of immunised mice**. The graph shows mean fluorescence intensities (MFI) obtained by flow-cytometric analysis of transfected and non-transfected HEK cells after staining with serum of mice (dilution 1:600) immunised with the corresponding cell line (PFF0620C-HEK, PFD1130W-HEK & PF14_0325-HEK). Values are means of two mouse sera and error-bars indicate the range.

The high-expressing cell pools PFF0620c-HEK, PFD1130W-HEK and PF14_0325-HEK were used to immunise NMRI mice. Mice received intravenous injections of 10^6 ^cells on three consecutive days and another suite of three daily injections two weeks later. Development of serum antibody titres was analysed by flow cytometry comparing immune-staining of the corresponding transfectants with that of non-transfected HEK cells (Figure 5). The fluorescence intensity observed with transfectants was two- to four-fold higher than that of non-transfected control HEK cells. This indicated that the mice had mounted an antibody response against the recombinant malaria antigens expressed on the surface of the transfected HEK cells.

**Figure 6:**
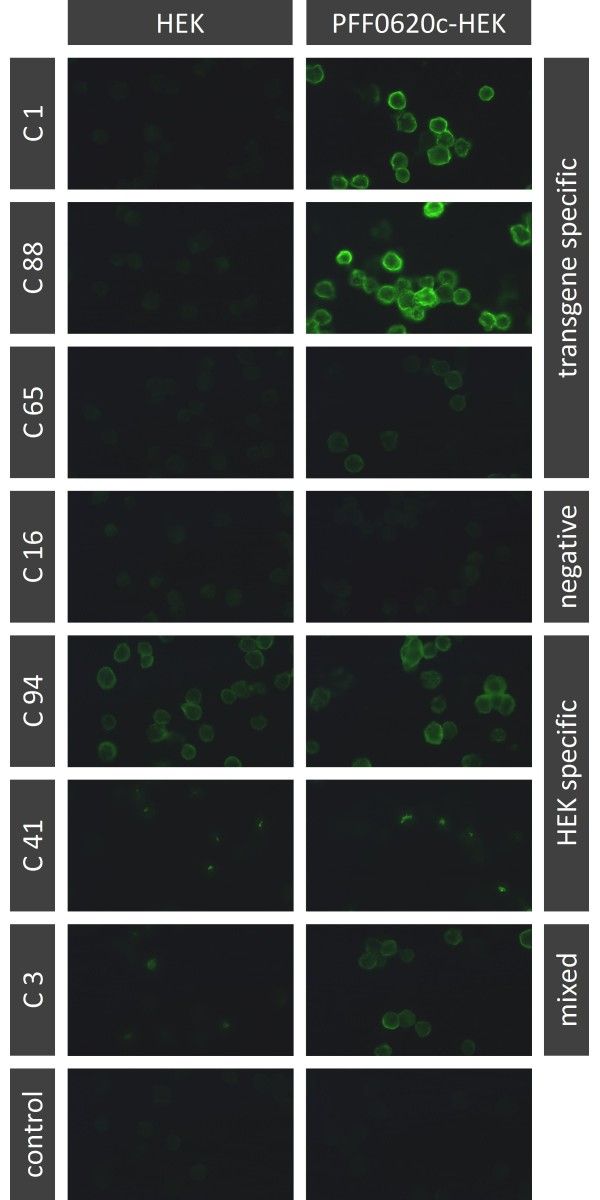
**Immunofluorescence microscopic screening of hybridoma cell culture supernatants for antibodies binding to cell-surface expressed PFF0620C**. PFF0620C-HEK cells (right column) and untransfected HEK cells (left column) were fixed with methanol and stained with hybridoma supernatants of individual cell culture wells taken 15 days after the fusion. A FITC-labelled anti-mouse IgG antibody served as secondary antibody. Representative fluorescence micrographs from the PFF0620C-fusion are shown, demonstrating typical results for hybidoma lines producing PFF0620C-specific (line 1, 2 & 3), non-binding (line 4), or HEK cell antigen specific (line 5 & 6) antibodies. For some wells mixed specificity was seen, indicating presence of two ore more cell clones with one producing Abs specific for the transgene and the other producing Abs specific for a HEK-cell protein (line 7).

Spleen cells of mice immunised with the transfected HEK cells were fused with PAI myeloma cells to generate B cell hybridomas. Fused cells were distributed in 1152 microtitre culture plate wells. To identify hybridoma cells that produce malaria antigen specific antibodies a two-step screening procedure was used that completely obviated the requirement for purified recombinant proteins. First all culture wells were tested for IgG production by ELISA. Between 18 and 29% of the tested wells were IgG positive (Table 1). In a second step all wells positive for IgG production were screened by IFA for antibodies binding to transfected cells. Transfected and non-transfected HEK cells spotted onto multiwell glass slides were stained with individual hybridoma supernatants and analysed by fluorescence microscopy (Figure 6). Non-transfected HEK cells served as a negative control for each sample. In all three fusions numerous culture supernatants positive on the transfected cells were also positive on non-transfected cells. However, each fusion yielded also numerous wells containing antibodies strongly reactive with transfectants but not reactive with untransfected HEK cells. Among these, eight wells were identified that contained antibodies reactive with an epitope present on all three transfectants but not on untransfected HEK cells. Most likely, these antibodies are specific for the FLAG tag or the membrane anchor domain. All other culture supernatants reactive with transfectants, but not with untransfected HEK cells were specific for the transfected cells used for immunisation and did not stain the two other transfectants (Table 1). From wells of this category, 17, nine and two hybridoma clones (Table 2) were derived by recloning from the PFF0620c, PFD1130W and PF14_0325-fusion, respectively.

**Table 1:** Specificity of hybridoma culture supernatants

	IgG ELISA screen^a^			IFA screen on transfected and non-transfected HEK cells^b^									
		
Antigen	total^c^	IgG positive^d^		total^e^		negative^f^		Pf protein specific^g^		HEK cell specific^h^		anchor specific^i^	
PFF0620c	1152	335	29%	142	42%	86	60%	25	18%	30	21%	1	0.7%
PFD1130w	1152	294	26%	294	100%	200	68%	10	3%	83	28%	1	0.3%
PF14_0325	1152	202	18%	202	100%	125	62%	2	1%	68	34%	7	3.0%

^a ^For each fusion hybridoma supernatants of 12 96-well-plates were analysed for the presence of mouse IgG by sandwich-ELISA

^b ^Wells screened positive by IgG ELISA were tested for binding to the corresponding transfected and the non-transfected HEK cells by IFA

^c ^Number of wells analysed by ELISA

^d ^Number and percentage of all tested wells that were IgG positive

^e ^Number and percentage of IgG positive wells that were analysed by IFA

^f ^Number and percentage of all wells tested by IFA that showed no reactivity in IFA

^g ^Number and percentage of all wells tested by IFA that only stained the transfectant cell line used for the immunisation of mice

^h ^Number and percentage of all wells tested by IFA that stained both transfected and non-transfected HEK cells by IFA

^i ^Number and percentage of all wells tested by IFA that stained all three transfectants, but were negative for non-transfected HEK cells

**Table 2:** Reactivity patterns of all generated mAbs with HEK cells and P. falciparum parasites

		Western blotting analysis				IFA			
			
Hybridoma Clone	Isotype	HEK	PFF0620c HEK	blood stages	sporozoites	HEK	PFF0620c HEK	blood stages	sporozoites
PFF0620c 01	IgG2a	-	+	-	-	-	+	-	+
PFF0620c 02	IgG2a	-	+	-	-	-	+	-	-
PFF0620c 06	IgG1	-	+	-	-	-	+	-	+
PFF0620c 07	IgG2a	-	+	-	-	-	+	-	+
PFF0620c 08	IgG1	-	+	-	-	-	+	-	-
PFF0620c 09	IgG1	-	+	-	-	-	+	-	+
PFF0620c 10	IgG1	-	+	-	-	-	+	-	+
PFF0620c 11	IgG1	-	+	-	-	-	+	-	+
PFF0620c 13	IgG2a	-	+	-	-	-	+	-	+
PFF0620c 17	IgG2b	-	+	-	-	-	+	-	+
PFF0620c 19	IgG1	-	+	-	-	-	+	-	-
PFF0620c 21	IgG2b	-	+	-	-	-	+	-	+
PFF0620c 22	IgG1	-	+	-	-	-	+	-	+
PFF0620c 25	IgG1	-	+	-	-	-	+	-	+
PFF0620c 27	IgG2b	-	+	-	-	-	+	-	-
PFF0620c 29	IgG1	-	+	-	-	-	+	-	+
PFF0620c 32	IgG1	-	-	-	-	-	+	-	+

		0/17	16/17	0/17	0/17	0/17	17/17	0/17	13/17
									
**Hybridoma** **Clone**	**Isotype**	**HEK**	**PFD1130w** **HEK**	**blood** **stages**	**sporozoites**	**HEK**	**PFD1130w** **HEK**	**blood** **stages**	**sporozoites**

PFD1130w 02	IgG2b	-	+	+	-	-	+	+	-
PFD1130w 04	IgG3	-	+	+	-	-	+	+	-
PFD1130w 05	IgG2a	-	+	+	-	-	+	+	-
PFD1130w 06	IgG2a	-	+	+	-	-	+	+	-
PFD1130w 08	IgG1	-	+	+	-	-	+	+	-
PFD1130w 09	IgG3	-	+	+	-	-	+	+	-
PFD1130w 10	IgG1	-	+	+	-	-	+	+	-
PFD1130w 12	IgG2a	-	+	+	-	-	+	+	-
PFD1130w 13	IgG1/IgG2b	-	+	+	-	-	+	+	-

		0/9	9/9	9/9	0/9	0/9	9/9	9/9	0/9
									
**Hybridoma** **Clone**	**Isotype**	**HEK**	**PF14_0325** **HEK**	**blood** **stages**	**sporozoites**	**HEK**	**PF14_0325** **HEK**	**blood** **stages**	**sporozoites**

PF14_0325 02	IgG1	-	+	+	nd	-	+	+	nd
PF14_0325 04	IgG1	-	+	+	nd	-	+	+	nd

		0/2	2/2	2/2		0/2	2/2	2/2	

nd = no data

**Figure 7:**
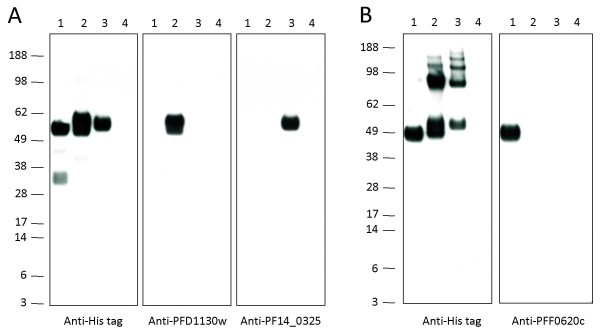
**Western blot analysis of generated monoclonal antibodies with the recombinant *P. falciparum *proteins**. Specificity of representative monoclonal antibodies for the corresponding recombinant proteins is demonstrated by Western-blot analysis. Reduced (A) and non-reduced (B) protein lysates of PFF0620c- (line 1), PFD1130w- (line 2), PF14_0325-expressing HEK cells (line 3) and non-transfected HEK cells (line 4) were probed with anti-His tag mAb, anti-PFD1130w 02 mAb, anti-PF14_0325 04 mAb and anti-PFF0620c 07 mAb, respectively.

The specificity of the monoclonal antibodies was further confirmed by Western blot analysis (Figure 7). With the exception of one anti-PFF0620c mAb, all 28 mAbs stained the corresponding recombinant protein in the lysate of the transfectant used for immunisation, but not in lysates of the other transfectants or of untransfected HEK cells (Table 2). Interestingly, in Western blot analysis anti-PFF0620c mAbs only bound to the non-reduced form of the recombinant protein. In contrast, anti-PFD1130w and anti-PF14_0325 mAbs bound both to the reduced and non-reduced recombinant proteins (data not shown).

### Reactivity of the generated monoclonal antibodies with the endogenous target antigens

**Figure 8:**
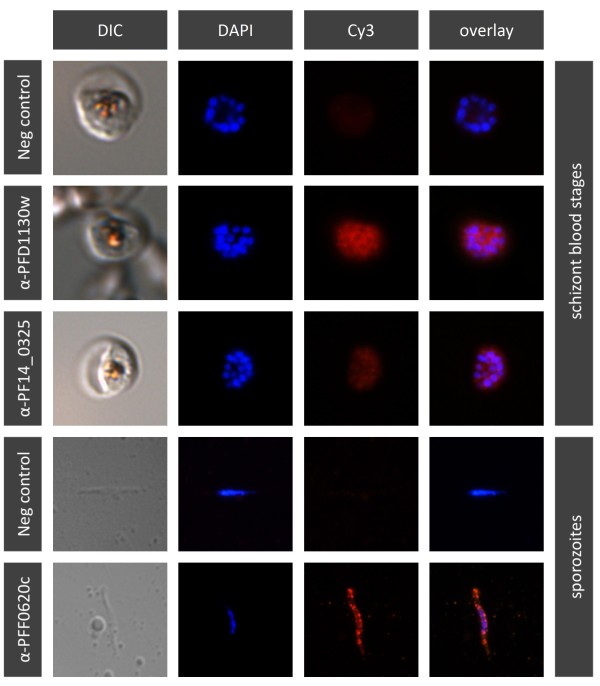
**Reactivity of generated monoclonal antibodies with the endogenous *P. falciparum *proteins**. Fluorescence staining (column 2, 3 & 4) and DIC micrographs (column 1) of *P. falciparum *parasites. While anti-PFF0620C mAb specifically stained salivary gland sporozoites (line 5), anti-PF14_0325 mAb (line 3) and anti-PFD1130W mAb (line 2) specifically reacted with schizont blood stage parasites. Staining with only the Cy3-labelled rabbit anti-mouse IgG secondary antibody served as negative control (line 1 & 4). Parasite nuclei were stained with DAPI.

To determine whether the generated mAbs selected against ectopically expressed PFF0620c, PFD1130W or PF14_0325 would bind to the endogenous proteins of *P. falciparum *parasites, we performed IFA and Western-blot analysis using cultivated blood-stage parasites and mosquito salivary gland-derived sporozoites (Figure 8 & Table 2). All nine generated anti-PFD1130W mAbs recognised a discrete band of about 40 kDa, corresponding to the predicted molecular weight of the PFD1130W protein. Furthermore all nine mAbs stained blood-stage parasites in IFA, resulting in a distinctive staining pattern (Figure 8 & Table 2). Both anti-PF14_0325 mAbs were Western-blot positive on blood stage parasite lysate and stained specifically late blood stage parasites in IFA (Figure 8 & Table 2). Thirteen of the seventeen mAbs specific for PFF0620c reacted in IFA with mosquito salivary gland sporozoites, but not with blood stage parasites in IFA (Figure 8 & Table 2). No reactivity was seen in Western blot with sporozoite lysate.

## Discussion

Membrane proteins, including membrane associated proteins, are the gatekeepers of the cell and selectively mediate the flow of information and nutrients between the outside and inside of the cell. Functions of membrane proteins include: transport of substances across membranes, enzymatic activity, signal transduction, intercellular joining, cell-cell recognition, attachment to the cytoskeleton and extracellular matrix. Even though only 25% of all proteins are membrane proteins, about 60% of today's approved drug targets are membrane proteins [15]. Also for subunit vaccine design cell surface associated proteins, which can be accessed by antibodies, are of prime interest. Nonetheless the basic knowledge about these proteins lags far behind that of soluble proteins. This is due to difficulties in ectopic expression, purification and protein stability. Therefore investigation of membrane proteins is a demanding undertaking and generation of mAbs against membrane proteins represents a difficult task.

Here we described a procedure to generate mAbs specific for cell-surface associated proteins, which completely bypasses any need for purified antigen. This is highly desirable as it supersedes the laborious task of antigen purification and ensures presentation of target proteins in their native conformation. In a study using antigen expressing transfectant cells to generate single chain antibodies it was shown that cell-based selection was far more efficient in generating Abs cross-reactive with the native endogenous protein than selection with purified recombinant protein [16].

The principal steps of the applied procedure are the following: (I) generation of stably transfected mammalian cells, expressing high levels of the target antigen on their surface in a native conformation; (II) immunisation of mice with transfected cells; (III) hybridoma cell generation; (IV) selection of hybridoma cells by a two step screening procedure involving an anti-IgG ELISA and an IFA with transfected and non-transfected cells. Only one single transfected cell line is required for the whole process including immunisation, serum analysis, screening of hybridoma cell lines and antibody characterisation. Furthermore the very same cell line may also be utilised for the study of receptor-ligand interactions.

In contrast to other entirely cell-based strategies to generate monoclonal antibodies [17,18,16] our procedure used a dual tagging strategy to be able to assess surface expression of the recombinant protein. In the case of all three transfectants, the His tag was only accessible upon fixation with methanol in IFA, whereas the FLAG tag was accessible on live cells. From these results we deduced that all three cell lines presented recombinant protein on their cell surface. Flow cytometric analysis showed that the staining intensity with the anti-FLAG tag and anti-His tag Ab was varying between the three different transfectants. The finding that transfectants showing the lowest staining with the anti-FLAG tag Ab showed the strongest staining with the anti-His tag Ab could indicate that the percentage of correctly orientated protein molecules is varying between the different transfectants. However, differences in accessibility of the two tags in the context of the different core proteins could also be the reason for these differences. Especially accessibility of the FLAG tag, which is positioned between the transmembrane domain and the parasite-derived part of the protein, could depend on the structural features of the parasite protein sequences.

In order to generate recombinant proteins with natural conformation, it is most favourable to use expression hosts of the same biological kingdom, since they likely produce the same post-translational modifications and recognise the same signals for synthesis, processing, and secretion as the organism, which the sequence was originally derived from. As we were dealing with *Plasmodium *proteins, this objective could not be fulfilled, because currently no Apicomplexa-based expression system is described. Anticipating surface expression and natural conformation, the protein encoding sequence was genetically modified in several ways. Firstly codon-usage was optimised for mammalian cells, as the plasmodium genome is extremely A+T-rich. The overall (A + T) composition is 80.6%, and rises to 90% in introns and intergenic regions [19]. Furthermore the endogenous GPI-attachment signal sequence was replaced by a transmembrane alpha helix of mouse glycophorin-A to ensure C-terminal membrane anchoring despite potential phylogenetic differences in the GPI-attachment machinery [20,21]. Whether recombinantly expressed proteins will contain the same posttranslational modifications as the endogenous protein was uncertain. *Plasmodium *species have undergone a process of gene loss that has removed much of their capacity to produce glycosylated proteins other than GPI-anchored proteins [22]. Therefore recombinant expression of *P. falciparum *proteins in mammalian cells takes a risk of generating glycosylated forms of the protein, which are not found in the parasite. Generated mAbs might therefore not be capable of recognising the unglycosylated endogenous protein. However, in the study presented here 24 out of 28 generated mAbs were capable of recognising the endogenous protein in IFA. Therefore potential differences in the glycosylation status of recombinant and endogenous protein were inconsequential for antigen-antibody interactions. Beside protein glycosylation, disulfide bonds play an important role in the folding and stability of proteins, especially for proteins secreted to the extracellular medium. PFF0620c is predicted to contain two s48/45 domains. These domains are known to contain six position-conserved cysteine residues, resulting in a conserved set of disulfide bonds [23,24]. Generated anti-PFF0620c mAbs only reacted with recombinant PFF0620c when analysed under non-reducing conditions, indicating that PFF0620c expressed in HEK cells contained disulfide bridges. As 13 out of 17 mAbs recognised endogenous PFF0620c in IFA, disulfide bridge formation in HEK cells is likely to have resulted in endogenous protein-like structures.

To our knowledge, the present study shows for the first time that antigen-expressing tranfectants of xenogenic origin can well be used for immunisation of mice to efficiently generate antigen-specific mAbs. In the described procedure we used a human derived cell line (HEK) for immunisation of mice. Usage of HEK cells has the advantage that they are easy to grow, transfect very readily and are capable of expressing large amounts of recombinant proteins [25]. In contrast, our attempts to express the three *Plasmodium *proteins in NIH/3T3 cells using a pcDNA3.1 or a pEF6 expression vector were not successful (data not shown). One might expect that mice immunised with cells of xenogenic origin generate an excess of antibodies against HEK cell derived antigen, thereby repressing antibody generation specific for the parasite proteins of interest. Indeed we saw strong antibody reactivity against HEK cells. However, this procedure generated also mAbs against the target antigens at high frequencies (Table 1). On the other hand one could speculate that immunisation with xenogenic transfectants possibly results in a stronger activation of the immune system than immunisation with allogenic cells. Interestingly, the percentage of transgene-specific clones generated in the three individual fusions correlated with the malaria antigen expression level of the corresponding transfected cells used for the immunisation of mice. This indicates that high expression levels of the target antigen are needed. It remains to be elucidated in more detail how expression strength and cell line origin affect the efficiency of mAb generation by this procedure. Anyhow, our results indicate that any kind of cell line, which expresses the antigen in decent amounts, can be used for the generation of mAbs by the described procedure.

The two-step screening procedure consisting of an IgG-ELISA followed by IFA of transfected cells was quick, and the readout straightforward. In other studies using antigen-expressing cells for immunisation and hybridoma selection, either hybridoma supernatants have been analysed by flow cytometry or a reporter cell line has been used [16,26]. These screening strategies represent alternatives to the two-step screening procedure described here. This would have the advantage that hybridoma supernatants are screened for reactivity to living transfectants and not against cells that had undergone a fixation processes. However, both for flow cytometric and for reporter cell analysis, antigen-expressing cells need to be available in appropriate quality and number over the extended and not entirely predictable time period when hybridomas are ready for primary and secondary screening. In contrast, the described IFA-screen can be performed with slides prepared prior to the fusion and stored at -80°C.

## Conclusions

These results demonstrate that our cell-based system of immunisation and hybridoma selection offers a rapid and efficient mean of obtaining mAbs reactive with the native form of membrane-associated proteins. The strategy may easily be extended to a wide range of cell-surface proteins.

## Methods

### Bacterial strains and media

*E. coli *strain Top10 (TOP10 Chemically Competent *E. coli *Cells, Invitrogen) was used for the amplification of plasmids. Bacteria were grown in LB medium containing 100 μg/ml ampicillin.

### Construction of plasmids and transformation

A double-stranded oligonucleotide encoding the secretion signal sequence of bee-venom melittin was ligated to NheI digested pcDNA3.1 (Invitrogen) resulting in plasmid pcDNA3.1_BVM. Mouse glycophorin-A mRNA was obtained from mouse bone marrow cells and cDNA generated by two-step reverse-transcription PCR using primers designed to amplify specifically the cDNA of the transmembrane encoding region of glycophorin-A. This sequence was then ligated to NotI digested pcDNA3.1_BVM resulting in plasmid pcDNA3.1_BVM_GP_His. The plasmid was constructed such that unique NheI and NotI sites were preserved in the multiple cloning site. Then plasmid pUC57 containing codon optimised synthetic genes encoding *P. falciparum *genes PF14_0325, PFD1130W or PFF0620C respectively that lack sequences for the secretion signal peptide and for the GPI-attachment signal peptide (Genscript), were ligated into NotI/NheI digested pcDNA3.1_BVM_GP_His. Thereafter a double-stranded oligonucleotide encoding a FLAG tag was ligated into the NotI site resulting in expression plasmids pcDNA3.1_BVM_PF14_0325_FLAG_GP_His, pcDNA3.1_BVM_PFD1130W_FLAG_GP_His and pcDNA3.1_BVM_PFF0620C_FLAG_GP_His.

### Culture of eukaryotic cells

The human embryonic kidney cell line 293 HEK was obtained from the American Type Culture Collection (CRL-1573, ATCC). 293 HEK cells were cultured in DMEM supplemented with 10% foetal calf serum, glutamine and penicillin/streptomycin at 37°C in a humidified incubator.

### Establishment of HEK 293 cell lines stably expressing PF14_0325, PFD1130W or PFF0620C

293 HEK cells were transfected with pcDNA3.1_BVM_PF14_0325_FLAG_GP_His, pcDNA3.1_BVM_PFD1130W_FLAG_GP_His and pcDNA3.1_BVM_PFF0620C_FLAG_GP_His respectively using JetPEI™(PolyPlus) transfection reagent. One day prior to transfection, a total of 5 × 10^5 ^293 HEK cells were seeded in 35-mm dishes. Transfection was performed following the manufacturer's protocol. 3 μg of expression plasmid and 6 μl transfection solution was used. Antibiotic selection was started 48 h after transfection. The selection medium containing 500 ug/ml of Geneticin (Gibco) was exchanged every 3-4 days.

### Generation of anti-FLAG tag and anti-His tag mAb

The mAbs His-6/9 and FLAG-27 were raised in Naval Medical Research Institute (NMRI) mice injected intraperitoneally with 20 μg of the respective peptides CGGHHHHH and CGGDYKDDDDL conjugated to KLH (Imject® Maleimide Activated mcKLH, Pierce) and emulsified in aluminum hydroxide gel (Alhydrogel-2%, Brenntag Biosector) containing CPG-OGN according to Davis et al 1998 [27]. The animals received up to four booster injections each at 3-week intervals with the same antigen preparation. As soon as the animals showed a specific immune response to the immunogen, the best responders were boosted and after 3 days, the spleens were removed and the isolated cells fused to PAI myeloma cells, a variant of the P3-x63-AG8 myeloma [1,28].

### Immunofluorescence staining of methanol-fixed HEK cells

HEK cells were collected using enzyme free dissociation buffer (Cell dissociation buffer enzyme-free Hanks'-based, Gibco), washed with PBS and spotted on multiwall glass slides (multitest slide, 12-well, 7 mm, ICN Biomedicals Inc.). When air-dried, cells were fixed in methanol for 10 min. Immunostaining was performed by incubating the wells with 30 μl of an appropriate mAb diluted in PBS or hybridoma culture supernatant for 20 min in a humid chamber at 37°C. After rinsing twice and washing for 15 min in PBS, 30 μl of 125 μg/ml FITC-conjugated goat anti-mouse IgG antibodies (RAM/IgG(H+L)/FITC, Nordic Immunological Laboratories) diluted in PBS were added to the wells and incubated for 20 min in a humid chamber at 37°C. Finally, slides were rinsed twice and washed for 15 min in PBS, mounted with mounting solution (50% PBS 50% glycerol) and covered with a coverslip. Stainings were assessed by fluorescence microscopy on a Leica CTR500 fluorescence microscope and a Leica DFC300 FX digital camera.

### Immunofluorescence staining of living HEK cells

For immunofluorescence staining of live HEK cells chamber slides (4-well chamber-slide, Lab-Tek™, Nunc™) were used. Wells were coated with 100 mg/l poly-D-lysine in H_2_O in a humid box at room temperature over night. After washing the wells three times with sterile H_2_O, 40'000 cells were seeded per well. Three days later the immunostaining was performed by incubating the wells with 500 μl of an appropriate mAb diluted in serum-free culture medium for 30 min on ice. After washing two times with serum-free culture medium 500 μl of 100 μg/ml FITC-conjugated goat anti-mouse IgG antibodies (RAM/IgG(H+L)/FITC, Nordic Immunological Laboratories) diluted in serum-free culture medium were added to the wells and incubated for 30 min on ice. Finally, the wells were rinsed twice with serum-free culture medium and once with D'PBS (Dulbecco's Phosphate-Buffered Saline containing calcium, Gibco). The slides were mounted with mounting solution containing DAPI (ProLong® Gold antifade reagent with DAPI, Invitrogen) and covered with a coverslip. Stainings were assessed as described above.

### Western Blotting analysis

HEK cells were collected using enzyme free dissociation buffer (Cell dissociation buffer enzyme-free Hanks'-based, Gibco), and washed two times with PBS. To prepare cell lysate, 10^6 ^cells were lysed with 0.1 ml of lysis buffer (1% NP40, 10% glycerol, 2 mM EDTA, 137 mM NaCl, 20 mM TrisHCl, pH8, Protease Inhibitors) for 10 min on ice. The lysate was cleared by centrifugation at 20'000 g for 5 min.

Blood stage parasite lysates were prepared essentially as described previously by saponin lysis of *P. falciparum *3D7-infected erythrocytes [29]. In brief, cultured parasites were washed once with PBS. Pelleted infected red blood cells were lysed by mixing with 20 volumes of 0.015% (w/v) saponin in PBS and incubated on ice for 20 min. Finally, the pelleted parasites were resuspended in 3 volumes of PBS and stored at -80°C until further use.

For SDS-PAGE cell- or parasite lysate was resolved on precast 4-12% gradient gels (NuPAGE® Novex 4-12% Bis-Tris Gel, Invitrogen) with MES running buffer according to the manufacturer's directions. The proteins were electrophoretically transferred to nitrocellulose membrane using a dry-blotting system (iBlot, Invitrogen). After blocking the membrane over night in blocking buffer (5% Milk in PBS) at 4°C, specific proteins were detected with appropriate dilutions of mAbs in blocking buffer for 1 h at room temperature. The membrane was then washed four times for 5 minutes in blocking buffer and incubated with horseradish peroxidase conjugated anti-mouse IgG mAb (GAM/IgG (γ-chain)/HRP) diluted 1:10'000 in blocking buffer at room temperature for 1 h. After washing again, blots were developed using ECL Western blotting detection reagents (ECL Western Blotting Substrate, Pierce) to visualise bands.

### FACS and Flow cytometric analysis of HEK cells

For sorting stably transfected cells into high-expressing cell pools, cells were dissociated with enzyme-free dissociation buffer (Cell dissociation buffer enzyme-free Hanks'-based, Gibco), washed with blocking buffer (PBS containing 3% BSA). The cells were then incubated with 200 μl of 100 μg/ml FLAG-27 mAb diluted in blocking buffer for 15 min on ice. The cells were then washed with blocking buffer and incubated with 200 μl of 100 μg/ml FITC-conjugated goat anti-mouse IgG antibodies (RAM/IgG(H+L)/FITC, Nordic Immunological Laboratories) diluted in blocking buffer for 15 min on ice. After a final wash the labelled cells were analysed and sorted using a Becton Dickinson FACSAria running Diva software. All analyses were performed using appropriate scatter gates to exclude cellular debris and aggregates. Gating settings were set to collect highly labelled cells. Post-sorting, the cells were collected in culture medium with 20% FCS and plated in 35 mm wells

For monitoring surface expression of PF14_0325, PFD1130W or PFF0620C on transfected cell lines, cells were stained as described above. FACS analysis was performed on a FACScan (Becton Dickinson) using CellQuest software (Becton Dickinson). 20'000 thousand events were collected for each sample. Untransfected cells served as negative control. For analysing seroconversion of immunised mice, transfected cell lines were stained using a 1:600 serum dilution in blocking buffer. Cells were staining and analysed as described above.

### Immunisation of mice

All procedures involving living animals were performed in accordance with the Rules and Regulations for the Protection of Animal Rights (Tierschutzverordnung) of the Swiss Bundesamt für Veterinärwesen. Naval Medical Research Institute (NMRI) mice were immunised by intravenous injections of 10^6 ^stably transfected HEK cells. Cells were thawed, washed and resuspended in 0.9% NaCl. Injections were accomplished on three consecutive days and after two weeks again on three consecutive days. Two to three weeks after the boost, blood was collected and the serum was tested for the presence of anti-PF14_0325, anti-PFD1130W or anti-PFF0620C antibodies, respectively by IFA and flow cytometry using stably transfected 293 HEK cells. Mice immunised with PF14_0325-HEK cells were boosted a second time after 4 weeks.

### Fusion and cell-based selection

Animals with serum strongly reactive with expressing cells were selected for fusion. These received a final injection of 10^6 ^cells two and one day before the fusion. Mice were sacrificed and the spleen was removed. Spleen cells were harvested by trituration under sterile conditions and fused with the myeloma cell partner (PAI mouse myeloma cells, derived from P3-x63-AG8) using polyethylene glycol 1500 (Roche Diagnostics). The fusion mix was plated into multiwell plates and hybridomas were selected by growing in HAT medium supplemented with culture supernatant of mouse macrophages P388. Wells were screened for specific IgG production between 2-3 weeks post-fusion by ELISA and IFA as described below. Cells from wells positive in initial screens were cloned by limiting dilution to obtain monoclonal populations.

### IgG ELISA screen

Maxisorp™plates (Nunc) were coated overnight at 4°C in a humid box with 100 μl of 5 μg/ml goat anti-mouse IgG (γ-chain specific) mAb (Sigma) diluted in PBS. After two washings with PBS containing 0.05% Tween-20, wells were blocked with blocking buffer (50 mM Tris, 140 mM NaCl, 5 mM EDTA, 0.05% NONidet P40, 0.25% gelatine, 1% BSA) for 1 h at 37°C and afterwards washed two times. 50 μl hybridoma supernatants were added to the wells and incubated for 1 h at 37°C. After washing 4 times, plates were incubated with 50 μl horseradish peroxidase-conjugated goat anti-mouse IgG (γ-chain specific) (Sigma) diluted 1:1000 in blocking buffer for 1 h at room-temperature in a humid box in the dark. After washing 4 times, TMB peroxidase substrate solution was added and the colour change monitored.

### Antibody production and characterisation

Identification of antibody subclasses was performed using a Mouse Monoclonal Antibody Isotyping Kit (ISO2, Sigma). For large-scale mAb production hybridoma cell lines were cultured in 500 ml roller-bottles (Corning). MAbs were purified by affinity chromatography using protein A or protein G Sepharose.

### *P. falciparum *blood stage culture

*P. falciparum *strain 3d7 was cultured essentially as described previously [29]. The culture medium was supplemented with 0.5% AlbuMAX (Gibco) as a substitute for human serum [30]. Cultures were synchronised by sorbitol treatment [31]. Erythrocytes for passages were obtained from the Swiss Red Cross (Switzerland).

### Immunofluorescence staining of *P. falciparum*

Erythrocytes from in vitro cultures of *P. falciparum *strain 3d7 were fixed in paraformaldehyde-glutaraldehyde as described previously [3]. Cells were blocked by incubation in 3% BSA in PBS for 1 h. Immunostaining was performed by incubation with an appropriate mAb dilution in blocking solution for 1 h. After three washes with PBS, 5 μg/ml cyanine dye (Cy3)-conjugated affinity-pure F(ab')2 fragment goat anti-mouse IgG (Fc-specific) antibodies (Jackson Immuno Research Laboratories) diluted in blocking solution was added and incubated for 1 h at RT. Finally cells were washed three times with PBS, mounted with mounting medium containing DAPI (ProLong® Gold antifade reagent with DAPI, Invitrogen) and covered with a coverslip. Antibody stainings were assessed as described above.

For immunfluorescence staining of sporozoites, air-dried unfixed *P. falciparum *(strain NF54) salivary gland sporozoites attached to microscope glass slides were incubated with mAbs and detected with cyanine dye (Cy3)-conjugated affinity-pure F(ab')2 fragment goat anti-mouse IgG (Fc-specific) antibodies (Jackson Immuno Research Laboratories) as previously described [32].

## List of abbreviations

Ab: antibody; Abs: antibodies; BSA: bovine serum albumin; DAPI: 4'-6-Diamidino-2-phenylindole; DIC: differential interference contrast; ELISA: Enzyme-linked Immunosorbent Assay; FACS: Fluorescence activated cell sorting; FCS: foetal calf serum; GPI: Glycosylphosphatidylinositol; HAT: Hypoxanthine Aminopterin Thymidine; HEK: Human Embryonic kidney cells; IFA: Immuno-fluorescence Assay; IgG: Immunoglobulin G; kDa: kilo Dalton; mAb: monoclonal antibody; mAbs: monoclonal antibodies; MFI: mean fluorescence intensity; PBS: phosphate-buffered saline; RT: room temperature

## Competing interests

The authors declare that they have no competing interests.

## Authors' contributions

AD was responsible for experimental design, performed the experiments and data analysis described in this study and drafted the manuscript. JB participated in the study design and designed the expression vectors. HM contributed to the conception of the study, participated in its design and assisted in data interpretation. GP contributed to the conception of the study, participated in the study design, coordinated the collaborations that made this study possible and revised the manuscript. All authors have read and approved the final manuscript.

## Supplementary Material

Additional file 1 **DNA and amino acid sequence of the antigen modifications**. Protein-coding DNA sequence and amino acid sequence of the modifications applied to the recombinant proteins, which allow for surface expression and detection of the recombinant protein. Click here for file

Additional file 2 **Protein-coding DNA sequence of the expression vectors**. Protein-coding DNA sequence of the expression vectors used to express the three different recombinant proteins on the surface of HEK cells. Click here for file

## References

[B1] Köhler G Milstein C Continuous cultures of fused cells secreting antibody of predefined specificity Nature 1975 256 495 497 10.1038/256495a0 1172191

[B2] Casadevall A Dadachova E Pirofski L Passive antibody therapy for infectious diseases Nat Rev Microbiol 2004 2 695 703 10.1038/nrmicro974 15372080

[B3] Breedveld FC Therapeutic monoclonal antibodies Lancet 2000 355 735 740 10.1016/S0140-6736(00)01034-5 10703815

[B4] Spangler BD Binding to native proteins by antipeptide monoclonal antibodies J Immunol 1991 146 1591 1595 1993848

[B5] Sahdev S Khattar SK Saini KS Production of active eukaryotic proteins through bacterial expression systems: a review of the existing biotechnology strategies Mol Cell Biochem 2008 307 249 264 10.1007/s11010-007-9603-6 17874175

[B6] Baneyx F Mujacic M Recombinant protein folding and misfolding in Escherichia coli Nat Biotechnol 2004 22 1399 1408 10.1038/nbt1029 15529165

[B7] Midgett CR Madden DR Breaking the bottleneck: eukaryotic membrane protein expression for high-resolution structural studies J Struct Biol 2007 160 265 274 10.1016/j.jsb.2007.07.001 17702603

[B8] Butler JE Navarro P Sun J Adsorption-induced antigenic changes and their significance in ELISA and immunological disorders Immunol Invest 1997 26 39 54 10.3109/08820139709048914 9037611

[B9] Snow RW Guerra CA Noor AM Myint HY Hay SI The global distribution of clinical episodes of Plasmodium falciparum malaria Nature 2005 434 214 217 10.1038/nature03342 15759000 PMC3128492

[B10] Gilson PR Nebl T Vukcevic D Moritz RL Sargeant T Speed TP Schofield L Crabb BS Identification and stoichiometry of glycosylphosphatidylinositol-anchored membrane proteins of the human malaria parasite Plasmodium falciparum Mol Cell Proteomics 2006 5 1286 1299 10.1074/mcp.M600035-MCP200 16603573

[B11] Bozdech Z Llinás M Pulliam BL Wong ED Zhu J DeRisi JL The transcriptome of the intraerythrocytic developmental cycle of Plasmodium falciparum PLoS Biol 2003 1 E5 10.1371/journal.pbio.0000005 12929205 PMC176545

[B12] Le Roch KG Zhou Y Blair PL Grainger M Moch JK Haynes JD De La Vega P Holder AA Batalov S Carucci DJ Winzeler EA Discovery of gene function by expression profiling of the malaria parasite life cycle Science 2003 301 1503 1508 10.1126/science.1087025 12893887

[B13] van Dijk MR van Schaijk BCL Khan SM van Dooren MW Ramesar J Kaczanowski S van Gemert GJ Kroeze H Stunnenberg HG Eling WM Sauerwein RW Waters AP Janse CJ Three members of the 6-cys protein family of Plasmodium play a role in gamete fertility PLoS Pathog 2010 6 e1000853 10.1371/journal.ppat.1000853 20386715 PMC2851734

[B14] Lasonder E Janse CJ van Gemert GJ Mair GR Vermunt AM Douradinha BG van Noort V Huynen MA Luty AJ Kroeze H Khan SM Sauerwein RW Waters AP Mann M Stunnenberg HG Proteomic profiling of Plasmodium sporozoite maturation identifies new proteins essential for parasite development and infectivity PLoS Pathog 2008 4 e1000195 10.1371/journal.ppat.1000195 18974882 PMC2570797

[B15] Yildirim MA Goh K Cusick ME Barabási A Vidal M Drug-target network Nat Biotechnol 2007 25 1119 1126 10.1038/nbt1338 17921997

[B16] Lipes BD Chen Y Ma H Staats HF Kenan DJ Gunn MD An entirely cell-based system to generate single-chain antibodies against cell surface receptors J Mol Biol 2008 379 261 272 10.1016/j.jmb.2008.03.072 18455737 PMC2496992

[B17] Spiller OB Harris CL Morgan BP Efficient generation of monoclonal antibodies against surface-expressed proteins by hyperexpression in rodent cells J Immunol Methods 1999 224 51 60 10.1016/S0022-1759(99)00008-3 10357206

[B18] Peipp M Simon N Loichinger A Baum W Mahr K Zunino SJ Fey GH An improved procedure for the generation of recombinant single-chain Fv antibody fragments reacting with human CD13 on intact cells J Immunol Methods 2001 251 161 176 10.1016/S0022-1759(01)00298-8 11292491 PMC7172470

[B19] Gardner MJ Hall N Fung E White O Berriman M Hyman RW Carlton JM Pain A Nelson KE Bowman S Paulsen IT James K Eisen JA Rutherford K Salzberg SL Craig A Kyes S Chan MS Nene V Shallom SJ Suh B Peterson J Angiuoli S Pertea M Allen J Selengut J Haft D Mather MW Vaidya AB Martin DM Fairlamb AH Fraunholz MJ Roos DS Ralph SA McFadden GI Cummings LM Subramanian GM Mungall C Venter JC Carucci DJ Hoffman SL Newbold C Davis RW Fraser CM Barrell B Genome sequence of the human malaria parasite Plasmodium falciparum Nature 2002 419 498 511 10.1038/nature01097 12368864 PMC3836256

[B20] Mendonça-Previato L Todeschini AR Heise N Previato JO Protozoan parasite-specific carbohydrate structures Curr Opin Struct Biol 2005 15 499 505 10.1016/j.sbi.2005.08.011 16154349

[B21] Delorenzi M Sexton A Shams-Eldin H Schwarz RT Speed T Schofield L Genes for glycosylphosphatidylinositol toxin biosynthesis in Plasmodium falciparum Infect Immun 2002 70 4510 4522 10.1128/IAI.70.8.4510-4522.2002 12117963 PMC128142

[B22] von Itzstein M Plebanski M Cooke BM Coppel RL Hot, sweet and sticky: the glycobiology of Plasmodium falciparum Trends Parasitol 2008 24 210 218 10.1016/j.pt.2008.02.007 18420458

[B23] Carter R Coulson A Bhatti S Taylor BJ Elliott JF Predicted disulfide-bonded structures for three uniquely related proteins of Plasmodium falciparum, Pfs230, Pfs48/45 and Pf12 Mol Biochem Parasitol 1995 71 203 210 10.1016/0166-6851(94)00054-Q 7477102

[B24] He X Grigg ME Boothroyd JC Garcia KC Structure of the immunodominant surface antigen from the Toxoplasma gondii SRS superfamily Nat Struct Biol 2002 9 606 611 12091874 10.1038/nsb819

[B25] Thomas P Smart TG HEK293 cell line: a vehicle for the expression of recombinant proteins J Pharmacol Toxicol Methods 2005 51 187 200 10.1016/j.vascn.2004.08.014 15862464

[B26] Mesci A Carlyle JR A rapid and efficient method for the generation and screening of monoclonal antibodies specific for cell surface antigens J Immunol Methods 2007 323 78 87 10.1016/j.jim.2007.02.007 17433358

[B27] McCluskie MJ Davis HL Cutting Edge: CpG DNA Is a Potent Enhancer of Systemic and Mucosal Immune Responses Against Hepatitis B Surface Antigen with Intranasal Administration to Mice J Immunol 1998 161 4463 4466 9794366

[B28] Stocker J Forster H Miggiano M Stahli C Staiger G Takacs B Staehelin T Generation of two new mouse myeloma cell lines PAI and PAI-0 for hybridoma production Res Disclos 1982 217 155 157

[B29] Matile H Pink JR Lefkovits I, Pernis B Plasmodium falciparum malaria parasite cultures and their use in immunology Immunological methods 1990 IV San Diego: Academic Press 221 234

[B30] Dorn A Stoffel R Matile H Bubendorf A Ridley RG Malarial haemozoin/beta-haematin supports haem polymerization in the absence of protein Nature 1995 374 269 271 10.1038/374269a0 7885447

[B31] Lambros C Vanderberg JP Synchronization of Plasmodium falciparum erythrocytic stages in culture J Parasitol 1979 65 418 420 10.2307/3280287 383936

[B32] Okitsu SL Kienzl U Moehle K Silvie O Peduzzi E Mueller MS Sauerwein RW Matile H Zurbriggen R Mazier D Robinson JA Pluschke G Structure-activity-based design of a synthetic malaria peptide eliciting sporozoite inhibitory antibodies in a virosomal formulation Chem Biol 2007 14 577 587 10.1016/j.chembiol.2007.04.008 17524988

