# Supplementary material for: An efficient system to generate monoclonal antibodies against membrane-associated proteins by immunisation with antigen-expressing mammalian cells
Source: BMC Biotechnol. 2010 Dec 15;10:87. doi: 10.1186/1472-6750-10-87 (PMC3019159; doi:10.1186/1472-6750-10-87)
Supplement: Additional file 1 — DNA and amino acid sequence of the antigen modifications. Protein-coding DNA sequence and amino acid sequence of the modifications applied to the recombinant proteins, which allow for surface expression and detection of the recombinant protein. [file 1472-6750-10-87-S1.PDF]

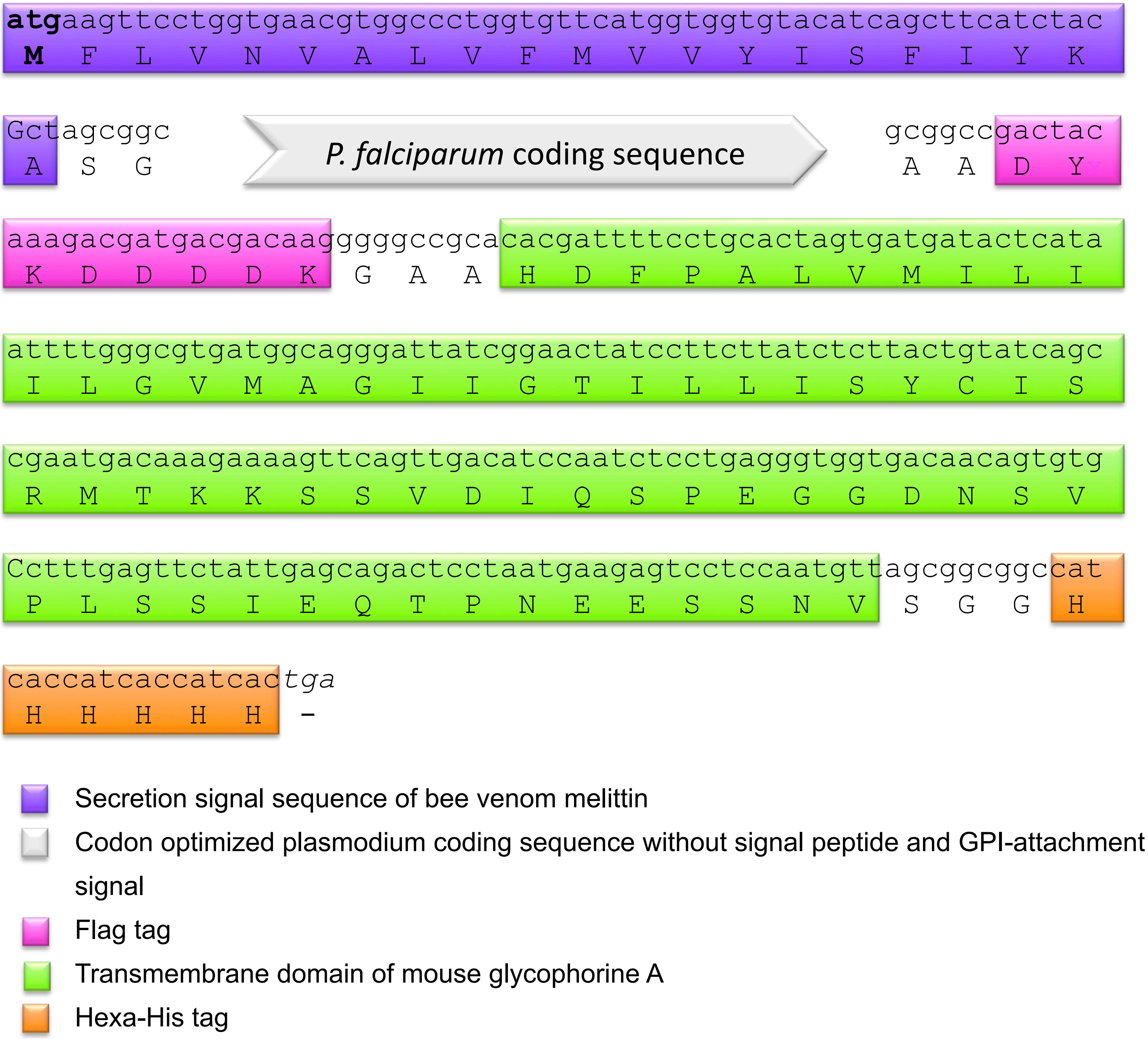

atgaagttcctggtgaacgtggccctggtgttcatggtggtgtacatcagcttcatctac
M F L V N V A L V F M V V Y I S F I Y K
Gctagcggc
A S G
P. falciparum coding sequence
gcggccgactac
A A D Y
aaagacgatgacgacaagggggccgcacacgattttcctgcactagtgatgatactcata
K D D D D K G A A H D F P A L V M I L I
attttgggcgtgatggcagggattatcggaactatccttcttatctcttactgtatcagc
I L G V M A G I I G T I L L I S Y C I S
cgaatgacaaagaaaagttcagttgacatccaatctcctgagggtggtgacaacagtgtg
R M T K K S S V D I Q S P E G G D N S V
Cctttgagttctattgagcagactcctaatgaagagtcctccaatgttagcggcggccat
P L S S I E Q T P N E E S S N V S G G H
caccatcaccatcactga
H H H H H -
Secretion signal sequence of bee venom melittin
Codon optimized plasmodium coding sequence without signal peptide and GPI-attachment signal
Flag tag
Transmembrane domain of mouse glycophorine A
Hexa-His tag
