# Supplementary material for: An efficient system to generate monoclonal antibodies against membrane-associated proteins by immunisation with antigen-expressing mammalian cells
Source: BMC Biotechnol. 2010 Dec 15;10:87. doi: 10.1186/1472-6750-10-87 (PMC3019159; doi:10.1186/1472-6750-10-87)
Supplement: Additional file 2 — Protein-coding DNA sequence of the expression vectors. Protein-coding DNA sequence of the expression vectors used to express the three different recombinant proteins on the surface of HEK cells. [file 1472-6750-10-87-S2.PDF]

PFD1130w

ATGAAGTTCCTGGTGAACGTGGCCCTGGTGTTCATGGTGGTGTACATCAGCTTCATCTACGCTAGCGGCATAAATTGTGACAGCCG
GCATGTGTTCATTAGAACCGAGTTGTCCTTCATAAAGAATAATGTGCCCTGCATTCGCGACATGTTCTTCATTTACAAGCGCGAGT
TGTACAACATCTGTTTGGATGACCTTAAGGGGGAAGAGGACGAGACCCATATCTACGTGCAGAAGAAGGTGAAGGACAGTTGGATA
ACCCTTAATGATCTGTTCAAAGAGACTGACTTGACTGGCAGACCTCACATCTTTGCCTACGTGGACGTGGAGGAGATCATCATCCT
GCTGTGTGAGGACGAAGAGTTTTCTAATCGTAAGAAGGATATGACTTGTCACAGGTTCTATTCAAATGACGGTAAGGAATACAACA
ACTCTGAAATCACTATAAGCGACTACATTCTCAAGGACAAGCTCTTGAGCTCCTACGTTAGCCTGCCCTTGAAGATTGAGAACCGG
GAGTACTTCCTCATCTGCGGGGTGAGCCCATACAAGTTTAAGGACGATAATAAGAAGGATGATATACTGTGTATGGCCTCCCACGA
CAAGGGAGAGACGTGGGGAACTAAGATAGTGATCAAGTATGATAATTACAAGCTCGGCGTGCAGTACTTCTTCCTCAGGCCTTACA
TCAGTAAGAACGACCTGTCCTTTCACTTTTATGTTGGAGACAACATCAATAATGTGAAAAACGTGAACTTTATAGAATGTACACAT
GAGAAAGACCTGGAGTTTGTCTGCTCCAATCGGGATTTCCTGAAAGACAACAAGGTGCTTCAGGACGTGAGCACGCTCAACGATGA
GTATATTGTGAGCTACGGCAACGACAACAACTTCGCTGAGTGTTACATCTTCTTCAACAATGAGAACAGCATCCTCATTAAGCCCG
AGAAGTATGGAAACACGACCGCCGGCTGTTACGGGGGAACCTTCGTGAAGATCGACGAAAATAGGACCCTGTTCATCTACTCTTCA
TCCCAGGGCATTTACAACGCGGCCGACTACAAAGACGATGACGACAAGGGGGCCGCACACGATTTTCCTGCACTAGTGATGATACT
CATAATTTTGGGCGTGATGGCAGGGATTATCGGAACTATCCTTCTTATCTCTTACTGTATCAGCCGAATGACAAAGAAAAGTTCAG
TTGACATCCAATCTCCTGAGGGTGGTGACAACAGTGTGCCTTTGAGTTCTATTGAGCAGACTCCTAATGAAGAGTCCTCCAATGTT
AGCGGCGGCCATCACCATCACCATCAC*TGA*

PF14_0325

ATGAAGTTCCTGGTGAACGTGGCCCTGGTGTTCATGGTGGTGTACATCAGCTTCATCTACGCTAGCGGCCACTTCCTTATCCGTTA
TAACCTGAACGGCGTGAGGTCTAACAACATCAGTCACAAGGAAGACGATTGCGGTGCCAGCGAGTTGGCTATCAAAAAGTTGCAGG
AGCTGAAGAAATTCGAGCTGGACATTCTGAAGGATTTCATTAAGAAGGACACCGACCACACTGACATGTACAAGAGGTATCACTGC
ATCAGCTCAGATTTCTTGAAGAACACAAAGCAGGAAGATAAGAAGGAAGACAATTACACCAAGAAGGAGGACAAAAAAGACATCGA
AAATTCCAGCAACTATATAATTAATAACAAAAATGAGAAGATCAGTTTCATAAACAAGATCATCTCATTTGTGGGGATGTGGAACT
ATAACAAGTACGGAAACAACATCCAGGAGAGTAAGAACAAGATCAACATCTACAACAACAACAACAATAAGACTGAGAATGAGCTG
CTTTACAACAAAGAGGACTACACAAATCATATTCCCACCAATGAAAATCCTAATTCCTACATGCAACTGTCCGCCTTCAACCTTAC
CAAAAAGAGTATGTTTCTCGGCGGAAAGGACAACGCCAGTCAGAACTTCGAAGTGGGCAATTACGGCTCTTTCTATATGATCTTTG
GAGCCCGGAATACAGACTACCCTTGGGCCTGTAGCTGCGATACACTCCAGCTGGAGGAATACAAAGAGAAGAAGCGAAGTTACGTG
CTGTGTTCAAATCAGCTCGACATGAGTATACAGAATAGCGATCTGTTTTGTAATCCTAAGAATGGGGCACACTACTATTACCTGTC
CGCGGCCGACTACAAAGACGATGACGACAAGGGGGCCGCACACGATTTTCCTGCACTAGTGATGATACTCATAATTTTGGGCGTGA
TGGCAGGGATTATCGGAACTATCCTTCTTATCTCTTACTGTATCAGCCGAATGACAAAGAAAAGTTCAGTTGACATCCAATCTCCT
GAGGGTGGTGACAACAGTGTGCCTTTGAGTTCTATTGAGCAGACTCCTAATGAAGAGTCCTCCAATGTTAGCGGCGGCCATCACCA
TCACCATCAC*TGA*

PFF0620c

ATGAAGTTCCTGGTGAACGTGGCCCTGGTGTTCATGGTGGTGTACATCAGCTTCATCTACGCTAGCGGCTATAAAATCAACGGCGT
CTGCGACTTTTCATCTGAAGGGCTGTCTCTGCTTCCAGAGGAGAAGCTGGATTTCTCTGTCAGTCGCAACGTTGATAAATTGTCCG
ACGAGAACAATGTGCGCCACTGTGTGCATTTTTCAAAGGGGTTCGAGTACCTGAGATTTATTTGCCCAATGCGGAAAGACAATTAC
GAGGGTATAGAGATTAGACCAGTGGAGTGTTTTGAGTACATTCATATCGAGGGGAGGGAGCACAAGTTGAGTGAGATTTTGAAGGG
CAGCCTGTATGAGAAGTCAATTAACGATAACATCATGACACGGGATGTCTTCATACCCCCCACGATTTATGAAGACATGTTCTTTG
AATGTACCTGCGACAATTCTCTTACCTTTAAGAACAATATGATTGGAATACGGGGTATTATGAAGATCCACCTGAAAAAGAACATT
CTCTATGGCTGCGACTTTGACCATGATGAGAAACTGATGAAGAACAAAACGGCCTTTACTAACTTCTACGATAAGCAGAAGATCCT
CCCCCTGATTGGCAACAACAATAACGATGATGATAACAACGACGATGACAACAACAACGACAACAACAATAACGACAACAATAATA
ACAATAACAACAACAACAACAATAACAACAATAATAATAATAACAACATCACGTGTAACGTTACCATCAAGAAAAGCCAGGTGTAT
CTGGGTATCATCTGCCCCGATGGATATACACTTTACCCAAATGATTGTTTTAAGAATGTGATCTACGACAATAACATCATCATTCC
TCTTAAGAAGATAATACCTCACGATATTCTCTATCATCAGGACAAAAATAAGAGGATTACCTTCGCCAGTTTTACCCTGAACATCA
ATGAAAACCCCCCCGGCTTCACCTGTTATTGTATCAAGGATCAGACAAATATCAATAATCCACTGATCGTGAATTTCCACTTCTCT
AACCAGGAAACCAGCTACGCCACAAAGAACAAGAATGCGGCCGACTACAAAGACGATGACGACAAGGGGGCCGCACACGATTTTCC
TGCACTAGTGATGATACTCATAATTTTGGGCGTGATGGCAGGGATTATCGGAACTATCCTTCTTATCTCTTACTGTATCAGCCGAA
TGACAAAGAAAAGTTCAGTTGACATCCAATCTCCTGAGGGTGGTGACAACAGTGTGCCTTTGAGTTCTATTGAGCAGACTCCTAAT
GAAGAGTCCTCCAATGTTAGCGGCGGCCATCACCATCACCATCAC*TGA*

Secretion signal peptide of bee venom melittin

Codon-optimized protein-coding sequence of *P. falciparum* gene without signal peptide and GPI-attachment signal sequence

Flag-tag

Transmembrane part of mouse glycophorine A

Hexa-His tag

Start codon

Stop codon
